# PI3K inhibition circumvents resistance to SHP2 blockade in metastatic triple-negative breast cancer

**DOI:** 10.1007/s10911-023-09539-9

**Published:** 2023-06-09

**Authors:** Romain J. Amante, Charly Jehanno, Duvini De Silva, Marie-May Coissieux, Markus Ackerknecht, Vincent Romanet, Atul Sethi, Baptiste Hamelin, Bogdan-Tiberius Preca, Salvatore Piscuoglio, Charlotte K. Y. Ng, Morvarid Mohseni, Mohamed Bentires-Alj

**Affiliations:** 1 Department of Biomedicine, University of Basel, University Hospital Basel, Basel, Switzerland; 2 Department of Surgery, University Hospital Basel, Basel, Switzerland; 3 Friedrich Miescher Institute for Biomedical Research, Basel, Switzerland; 4 Novartis Institutes for Biomedical Research, Disease Area Oncology, Basel, Switzerland; 5 Institute of Medical Genetics and Pathology, University Hospital Basel, Basel, Switzerland; 6 Department for BioMedical Research (DBMR), University of Bern, Bern, Switzerland; 7 Novartis Institutes for Biomedical Research, Disease Area Oncology, Cambridge, USA

**Keywords:** Breast cancer, Metastasis, PDGFRβ, SHP2, PI3K

## Abstract

The protein tyrosine phosphatase SHP2 activates oncogenic pathways downstream of most receptor tyrosine kinases (RTK) and has been implicated in various cancer types, including the highly aggressive subtype of triple-negative breast cancer (TNBC). Although allosteric inhibitors of SHP2 have been developed and are currently being evaluated in clinical trials, neither the mechanisms of the resistance to these agents, nor the means to circumvent such resistance have been clearly defined. The PI3K signaling pathway is also hyperactivated in breast cancer and contributes to resistance to anticancer therapies. When PI3K is inhibited, resistance also develops for example via activation of RTKs. We therefore assessed the effect of targeting PI3K and SHP2 alone or in combination in preclinical models of metastatic TNBC. In addition to the beneficial inhibitory effects of SHP2 alone, dual PI3K/SHP2 treatment decreased primary tumor growth synergistically, blocked the formation of lung metastases, and increased survival in preclinical models. Mechanistically, transcriptome and phospho-proteome analyses revealed that resistance to SHP2 inhibition is mediated by PDGFRβ-evoked activation of PI3K signaling. Altogether, our data provide a rationale for co-targeting of SHP2 and PI3K in metastatic TNBC.

**Supplementary Information:**

The online version contains supplementary material available at 10.1007/s10911-023-09539-9.

## Introduction

Breast cancer is the leading cause of cancer death among women. In 2020, 2.1 million new cases and 685,000 deaths were reported globally by the World Health Organization. One third of breast cancers progress to metastasis [1, 2], which is the cause of most breast cancer-related deaths. Metastasis is fueled by organ-specific feed-forward loops between cancer cells and the tumor microenvironment [3]. Thus, anti-cancer target identification and the development and testing of drugs should focus on metastatic cells before and/or after they have proliferated and spread to distant organs. This is key to the improvement of current therapies.

Src-homology 2 domain-containing phosphatase (SHP2), a ubiquitously expressed protein tyrosine phosphatase (PTP), transduces mitogenic, survival, cell-fate and/or migratory signals [4, 5] downstream of various active receptor tyrosine kinases (RTK). SHP2 is fundamental to the activation of the mitogen-activated protein kinase (MAPK)/extracellular signal-related kinase (ERK) pathway [6]. Germline-activating mutations of SHP2 cause Noonan syndrome [7] and somatic gain-of-function mutations prompt several hematological malignancies [8]. While *PTPN11* (Protein Tyrosine Phosphatase Non-Receptor Type 11) is rarely mutated in solid tumors, SHP2 is activated downstream of several oncogenic signals [9, 10]. Notably, small-hairpin knockdown of SHP2 decreases breast tumor growth and progression [4, 5, 11, 12]. Potent orally active allosteric inhibitors of SHP2 have been reported [13] and are currently being evaluated in phases I/II trials that focus on advanced solid tumors, mostly in non-small cell lung cancer (NSCLC), squamous cell carcinoma (SCC) or colorectal cancer (CRC) [14] (NCT03114319). While it was reported recently that SHP2 blockade enhances anti-tumor immunity [15], the effects of pharmacological inhibition of SHP2 on breast cancer metastasis and survival remain ill-defined. Moreover, the exact mechanisms of resistance to SHP2 inhibition are yet to be fully understood.

The phosphatidylinositol 3-kinase (PI3K) signaling axis is essential for cell survival, proliferation, motility and apoptosis [16]. Downstream of RTKs and G protein-coupled receptors, class I PI3Ks phosphorylate phosphatidylinositol-4,5-bisphosphate, generating phosphatidylinositol-3,4,5-triphosphate and leading to the activation of numerous kinases such as PDK1 (phosphoinositide-dependent kinase 1), AKT (also called protein kinase B), and p70 ribosomal protein S6 kinase (S6K) [17]. The PI3K pathway is one of the most frequently activated in human cancers, including 70% of breast cancers, and influences tumor initiation, progression, and resistance to therapy [18, 19]. Different classes of inhibitors targeting several key components of this pathway (*e.g*., PI3K, AKT, mTOR) have been developed over the last two decades, but their efficacy is limited by various mechanisms of resistance, including the activation of RTKs [16].

Given that PI3K activation contributes to resistance to anticancer agents, that RTKs often blunt the response to PI3K inhibition, and that SHP2 transduces oncogenic signaling downstream of most RTKs, we asked whether co-targeting these pathways would be more effective than single agents in preclinical models of metastatic TNBC. Using in vitro and in vivo models in both immunocompetent and immunodeficient mice, we compared the efficacy of PI3K and SHP2 inhibitors alone or in combination, and measured their impact on primary tumor and lung metastatic growth, as well as on animal survival. Here, we provide evidence that PI3K/SHP2 dual-inhibition reduces lung metastases and prolongs overall survival in preclinical models of metastatic TNBC.

## Results

### SHP2/PI3K dual-inhibition decreases cell number, reduces primary tumor growth, and increases overall survival in TNBC models

**Fig. 1:**
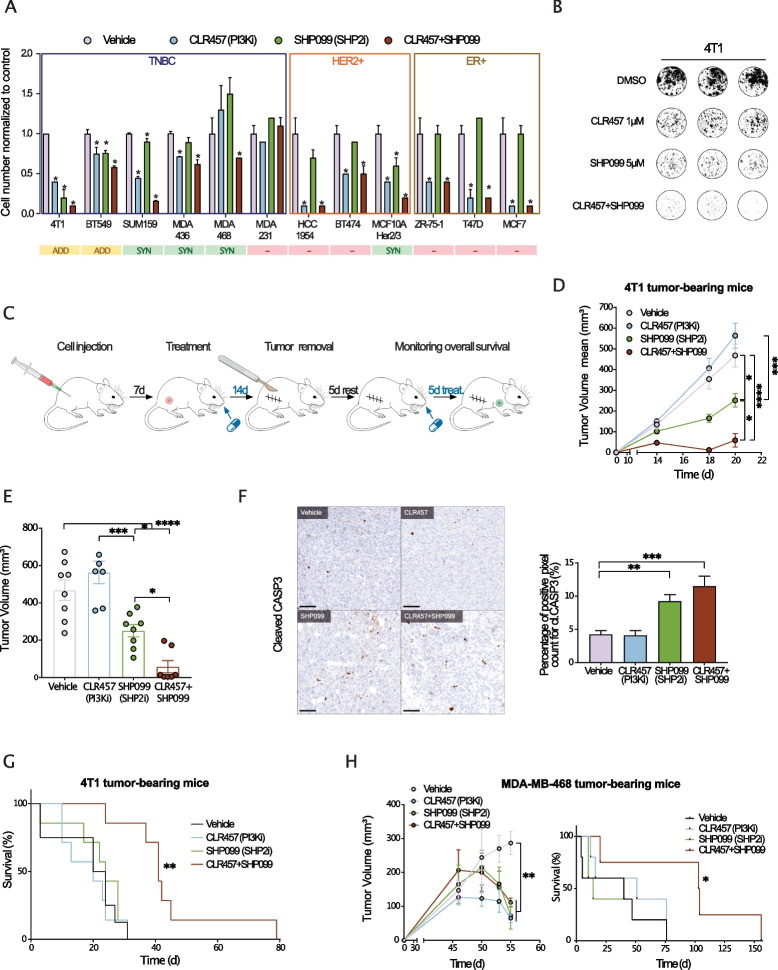
PI3K/SHP2 inhibition reduces TNBC cell number, primary tumor volume and metastasis with prolonged animal survival. **A** Cell numbers of breast cancer lines treated with CLR457 (PI3Ki, 2.5 µM) and/or SHP099 (SHP2i, 5 µM) for 72 h. Dual inhibition is compared to single inhibition in terms of additive or synergistic effect (ADD: Additive; SYN: Synergistic). *n* = 3 biological replicates. * *P* ≤ 0.05; One-way ANOVA test. Data shown are mean cell numbers ± STDEV. **B** Representative images of 4T1 cells treated with CLR457 (PI3Ki) and/or SHP099 (SHP2i) at the indicated concentrations for 72 h. Each condition is shown in triplicates. **C** Design of treatments in the neo-adjuvant setting. One week after tumor cell injection, treatments were applied for 14 days (d = days). Tumor removal and 5 days of recovery were followed by a second round of treatment of 5 days. The overall survival of the animals was monitored during the experiments. **D** Tumor volumes of 4T1 tumor-bearing mice treated with Vehicle, CLR457 and/or SHP099. *n* = 6 – 8 animals. * *P* ≤ 0.05, *** *P* ≤ 0.001, **** *P* ≤ 0.0001; One-way ANOVA test. Data shown are mean tumor volumes ± SEM. **E** Tumor volumes of 4T1 tumor-bearing mice after 14 days of treatment as indicated. *n* = 6 – 8 animals. * *P* ≤ 0.05, *** *P* ≤ 0.001, **** *P* ≤ 0.0001; One-way ANOVA test. Data shown are mean tumor volumes ± SEM. **F** Representative images of cleaved-Caspase 3 IHC staining of lung metastases from 4T1 tumor-bearing mice treated in the adjuvant setting for 4 days as indicated (left). Bar graph of the quantification using a pixel count algorithm performed with Halo software (right). Scale bar 100 µm. *n* = 3 animals from the same cohort. ** *P* ≤ 0.01, *** *P* ≤ 0.001; One-way ANOVA test. Data shown are means ± STDEV. **G** Overall survival of 4T1 tumor-bearing mice treated as indicated. An event was scored at the appearance of any sign of distress. *n* = 6 – 8 animals. ** *P* ≤ 0.001; Log-rank test. **H** Tumor volume and overall survival of MDA-MB-436 tumor-bearing mice treated as described (adjuvant settings). *n* = 4 – 6 animals. * *P* ≤ 0.05, ** *P* ≤ 0.01; One-way ANOVA test. Data shown are mean tumor volumes ± SEM

We first assessed the effects of the allosteric SHP2 inhibitor SHP099 [13] and/or the pan-PI3K inhibitor CLR457 [20] on the 4T1 mouse mammary carcinoma cell line and on a panel of human breast cancer cell lines. To better comprehend what dictates the response to these two targeted therapies, we established their mutational profile for key oncogenes and tumor suppressors (Fig S1A), as well as their baseline phosphorylation level for three major kinases p-ERK (T202/Y204, MAPK pathway), p-AKT (S473, PI3K pathway) and p-S6 (S235/S236, mTOR pathway) (Fig S1B, C). Our results show that TNBC cell lines specifically are more sensitive to dual PI3K and SHP2 inhibition (PI3Ki/SHP2i) than single inhibition, as compared to human epidermal growth factor receptor 2 (HER2)-enriched and estrogen receptor positive (ER +) cell lines (Fig. 1A). Indeed, all the TNBC lines except MDA-MB-231 (dual KRAS/BRAF mutant with high levels of pERK, Fig S1A-C) were more sensitive to dual inhibition, with three lines out of six showing a synergistic effect (SUM159, MDA-MB-436, MDA-MB-468) and two showing an additive effect (4T1, BT549) (Fig. 1A, Fig S2A-C). While PI3K inhibition (PI3Ki) alone did not induce apoptosis in 4T1 cells, SHP2 inhibition (SHP2i) induced apoptosis dramatically and the combination induced it even further (Fig. 1B, Fig S2D, E). Together, these results highlight the relevance of PI3K/SHP2 dual-inhibition in TNBC models.

To elucidate the effect of PI3K and SHP2 single or dual-inhibition in vivo, we first used the 4T1 syngeneic mouse model of metastatic breast cancer [21, 22] (Fig. 1C). Neoadjuvant PI3Ki had no effect on tumor growth, whereas SHP2i reduced average primary tumor volume by half. Strikingly, we observed that dual-inhibition had a synergistic effect and dramatically decreased primary tumor growth (Fig. 1D, E). Quantification of cleaved-Caspase 3 in tumors after treatment revealed increased apoptosis upon SHP2i and dual-inhibition (Fig. 1F).

Next, we addressed the relevance of PI3K/SHP2 dual-inhibition for treating metastatic disease. After tumor resection, mice injected with 4T1 cells were treated for 5 days in a second round of inhibition, and monitored until signs of distress appeared (Fig. 1C). Only dual PI3Ki/SHP2i enhanced overall survival, with a median survival of 41 days, as compared to Vehicle or single agent-treated mice, with a median survival of 20–24 days (Fig. 1G). We then evaluated whether PI3K and SHP2 co-targeting prolongs survival in immunocompromised mice orthotopically injected with the human TNBC cell lines MDA-MB-468 or MDA-MB-436. Similarly, we found that PI3Ki, SHP2i and dual-inhibition reduced primary tumor growth, however only the PI3K/SHP2 co-targeting prolonged animal survival, indicating that only the combination decreased metastatic burden (Fig. 1H, Fig S2F). Indeed, histological analysis of H&E-stained lung sections revealed the absence of 4T1 metastatic foci in CLR457/SHP099 treated animals (sacrificed at the end of the 5-day treatment) as compared to Vehicle and PI3K single inhibition (Fig S2G). Interestingly, animals after SHP2 single inhibition had no lung metastases as well (Fig S2G). Given that 4T1 cells form metastases in multiple organs, we speculate that mice may have died from metastases at a different site or that cessation of SHP2 single inhibition triggered an overshoot of metastasis, a phenomenon previously observed in another context [23] and prevented by dual PI3K/SHP2 inhibition (Fig S2G). Of note, size-based quantification revealed the presence of larger metastases in the PI3K-treated group than in the control, similar to our previous observation with TNBC models (MDA-MB-231 LM2, 4T1) (Fig S2H) [24]. Altogether, our data showed a clear benefit of PI3K/SHP2 dual inhibition for overall survival in metastatic TNBC.

### Re-activation of PI3K and ERK/MAPK signaling blunts the effects of SHP2 inhibition

**Fig. 2:**
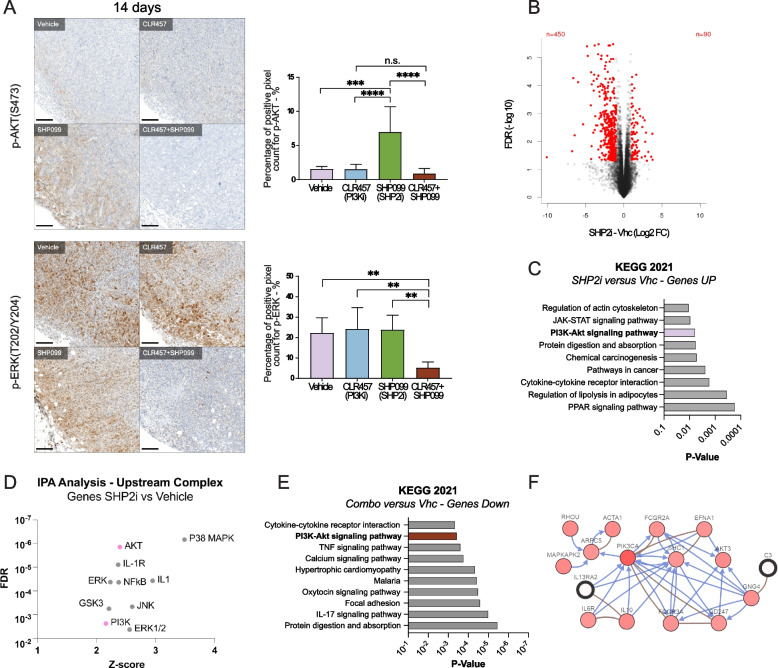
SHP2 blockade reactivates the PI3K pathway. **A** Representative images of the immunohistochemistry staining of 4T1 tumors from mice treated for 14 days with Vehicle, CLR457 and/or SHP099. Bar graphs show the quantification of p-AKT (Ser473) (top panel) and p-ERK (Thr 202/Tyr 204) staining (bottom panel). Tumors were collected as described in Fig. 1G. *n* = 7 – 8 animals. ** *P* ≤ 0.01, *** *P* ≤ 0.001, **** *P* ≤ 0.0001, n.s. not significant; One-way ANOVA test. Data are means ± STDEV. Scale bar 100 µm. **B** Volcano plot showing differentially expressed genes in SHP2i *versus* Vhc contrast in 4T1 tumors from mice treated as indicated. *n* = 4 – 5 animals, LogFC > 1, FDR < 0.05. **C** KEGG 2021 functional annotation of genes down-regulated upon SHP2i as compared to Vehicle. LogFC < -1, FDR < 0.05. **D** Upstream Regulator Analysis (Complex) from Ingenuity Pathway Analysis (IPA) of genes down-regulated upon SHP2i as compared to Vehicle. Pink dots represent AKT and PI3K complexes. LogFC < -1, FDR < 0.05. **E** KEGG 2021 functional annotation of genes down-regulated upon combo treatment as compared to Vehicle. LogFC < -1, FDR < 0.05. **F** Network generated using cBioportal showing the top 35 genes described in Fig S3G and their most frequently altered neighboring genes (filtered, 21%) in the breast cancer METABRIC dataset (Blue: control change of state; brown: in complex)

To decipher the molecular mechanisms by which PI3K/SHP2 dual inhibition decreases cancer cell viability, we assessed the short- and long-term effects of SHP2i and/or PI3Ki on the ERK/MAPK and PI3K pathways in vivo. Immunohistochemistry of 4T1 primary tumors after 4 or 14 days of treatment showed low levels of p-AKT (S473) in tumors from the Vehicle or PI3Ki-treated groups, regardless of treatment duration (Fig. 2A, Fig S3A). Interestingly, prolonged single SHP2i significantly increased p-AKT, indicating a reactivation of the PI3K kinase pathway in cancer cells upon SHP099 treatment. Dual inhibition blunted the activation of p-AKT upon SHP2i (Fig. 2A upper panel). SHP2i reduced p-ERK (T202/Y204) after 4 days (Fig S3A) but not after 14 days of treatment, where cells displayed a level of p-ERK similar to the control and to PI3Ki-treated samples (Fig. 2A bottom panel). Of note, the PI3K/SHP2 combination drastically reduced the level of p-ERK after 14 days of treatment (Fig. 2A bottom panel). Together, these results showed that both the PI3K and the ERK/MAPK pathways were reactivated in cells that survived single SHP2i, an effect that was prevented by PI3K/SHP2 dual inhibition.

### SHP2 inhibition converges on the PI3K pathway at the transcriptional level

Next, we assessed the in vivo effects of PI3K and/or SHP2 inhibition on the transcriptome of 4T1 tumors, after 14 days of treatment. First, principal component analysis (PCA) showed that the gene changes were similar within treatment groups and confirm the robustness of the dataset (Fig S3B). After PI3Ki treatment, 260 genes were up- or downregulated, 540 genes after SHP2i treatment, and 1531 after dual inhibition, using an FDR < 0.05 and an absolute LogFC of 1 (Fig. 2B, Fig S3E, F). Functional annotation using different resources (KEGG, Ingenuity Pathway Analysis, WikiPathways and GSEA) revealed PI3K/AKT signaling as a term consistently associated to genes upregulated upon SHP2i, and to genes downregulated upon PI3Ki (Fig. 2C-E, Fig S3C, D). Finally, network analysis of the 35 most-upregulated genes in the SHP2 inhibition group compared to the Vehicle group using cBioPortal (https://www.cbioportal.org/) and the breast cancer METABRIC dataset showed convergence to Src Homology 2 Domain-Containing 1 (SHC1), PIK3CA and AKT3 (Fig. 2F, Fig S3G). Altogether the data inferred from transcriptomic analyses are consistent with IHC results, and demonstrate reactivation of PI3K signaling upon SHP2i.

### PDGFRβ mediates reactivation of the PI3K pathway following SHP2i

**Fig. 3:**
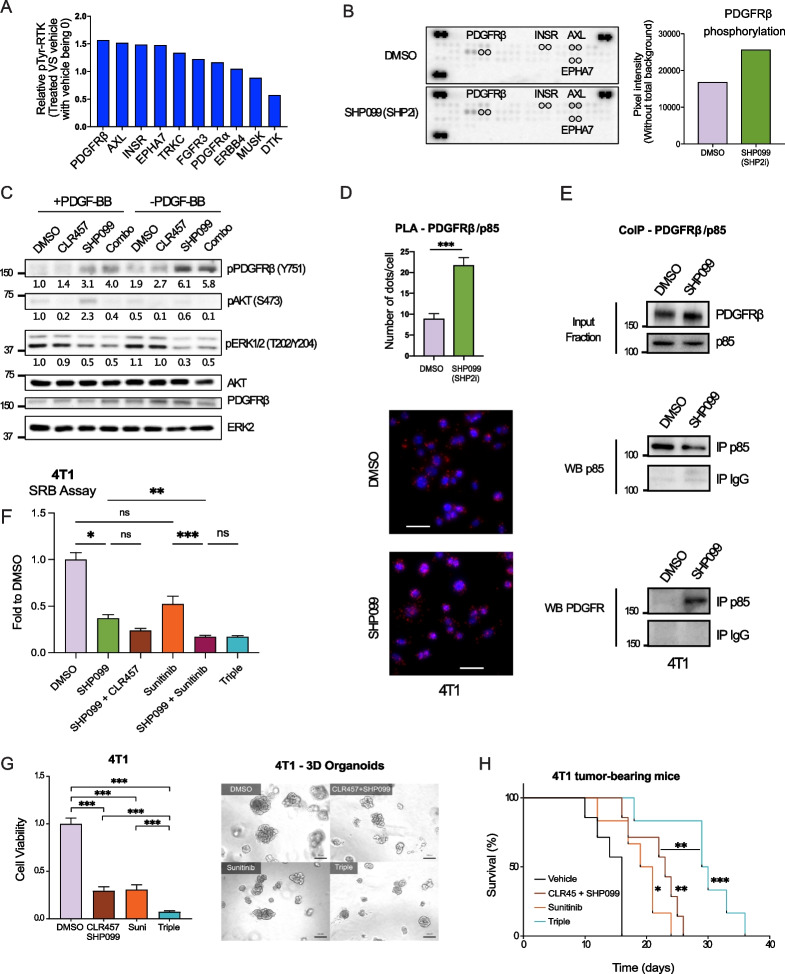
SHP2i induces PDGFRβ phosphorylation and subsequent PI3K pathway signaling. **A** Quantification of the 10 most-enriched phospho-tyrosyl-RTKs in 4T1 tumors from mice treated as indicated. Data shown are dot quantification by pixel density from RTK-array scans. **B** Receptor Tyrosine Kinase (RTK) array from 4T1 tumors of mice treated with Vehicle or SHP099 and bar graphs (right) of PDGFRβ phosphorylation. n = 1 RTK array. Data shown are dot quantification by pixel density from RTK-array scans. **C** Immunoblots of lysates from 4T1 cells that were grown as monolayers and treated with PI3Ki (CLR457, 500 nM) and/or SHP2i (SHP099, 5 µM) and stimulated with PDGF-BB 500 ng/mL for 20 h. n = 2 replicates. **D** Proximity Ligation Assay of PDGFRβ/p85 in 4T1 cells cultured in DMEM 1% FCS treated with DMSO or SHP2i (SHP099, 5 µM). Quantification of dots (interactions) per cell and representative images are shown. Brightness and contrast for the red and blue channels have been adjusted identically for all images. *n* = 3 biological replicates. *** *P* < 0.001; Student’s t-test. Error bars represent standard deviations. Scale bar 20 µM. **E** Immunoblots showing PDGFRβ co-immunoprecipitation with p85 in 4T1 cells treated with SHP099 (5 µM) for 24 h, as compared to DMSO treated cells. IgGs were used as negative control for p85 immunoprecipitation. PDGFRβ and p85 abundance in the input fraction are shown. **F** SRB assay using the 4T1 cells treated with DMSO, SHP099 (5 µM), CLR457 (2 µM), Sunitinib (0.5 µM) and the indicated combinations, for 3 consecutive days. n = 2 biological replicates with 4 technical replicates. * *P* ≤ 0.05, *** *P* ≤ 0.001; One-way ANOVA test. Data shown are mean ± STDEV. **G** 3D cell viability assay of 4T1 organoids treated as indicated for 4 days (left panel). Treatment was refreshed every 2 days. Representative images of 3D cultures from each condition are shown (right panel). *n* = 4 replicates. *** *P* ≤ 0.001; One-way ANOVA test. Data shown are means ± STDEV. Scale bar 100 µm. **H** Overall survival of 4T1-tumor-bearing mice treated in the adjuvant setting as indicated. An event was scored when a mouse showed any sign of distress. *n* = 7 – 8 animals per group. * *P* = 0.0111, ** *P* = 0.0015; *** *P* = 0.0009; Log-rank test

To identify molecular mechanisms accounting for SHP2i-mediated PI3K pathway activation, we quantified the total tyrosyl-phosphorylation of 39 RTKs in tumor protein lysates from SHP2i-treated tumors (Fig. 3A, B). Several RTKs were highly phosphorylated upon SHP2 inhibition, compared to the control, including platelet-derived growth factor receptor β (top hit; PDGFRβ), AXL, insulin-receptor (INSR) and ephrin-A7 (EPHA7) (Fig. 3A, B). Interestingly, PDGFRβ is known to promote tumor growth, metastasis, and resistance to therapy [25, 26]. We then asked whether SHP2i-mediated PDGFRβ hyper-phosphorylation accounts for subsequent PI3K pathway activation.

We first confirmed the absence of differential expression of PDGF receptors and ligands in all conditions, thus excluding the possibility of transcriptional mechanisms (Fig S4A-B). We next assessed the abundance of p-AKT (S473), p-ERK (T202/Y204) and p-PDGFRβ (Y751) by western blot in 4T1 cells treated in vitro. We show that SHP2 inhibition decreases the level of p-ERK and increases the level of p-AKT, which was blocked upon addition of PI3K inhibitor (Fig. 3C), therefore mirroring what we detected in vivo (Fig. 2A). Interestingly, SHP2i increased phosphorylation of PDGFRβ Y751, a substrate of SHP2 and a known docking site of the regulatory subunit of PI3K p85 (Fig. 3C) [27, 28]. We validated this observation in the human triple negative cell line MD-MDA-468, in which SHP2i also triggers PDGFRβ phosphorylation at Y751 (Fig S4C). To test whether PDGFRβ Y751 hyperphosphorylation upon SHP2i promotes recruitment of PI3K p85, we performed a proximity ligation assay (PLA), a technique that allows quantification of protein interactions. SHP2i resulted in a 1.5 to 2-fold increase in interactions between PDGFRβ and p85 compared to DMSO-treated 4T1 and MDA-MB-468 cells (Fig. 3D, Fig S4D). These results were confirmed using co-immunoprecipitation of PDGFRβ together with p85, specifically in SHP099-treated 4T1 cells compared to DMSO (Fig. 3E). These data indicate that SHP2i increases PDGFRβ Y751 phosphorylation and facilitates recruitment of the PI3K p85 subunit, which accounts for the subsequent activation of the PI3K pathway. Next, we assessed the effect of PDGFRβ inhibition alone or with SHP2i or PI3Ki/SHP2i dual inhibition, both in vitro and in vivo, using PDGFRβ inhibitor Sunitinib [29]. Sunitinib treatment with SHP2i and SHP2i/PI3K decreased 4T1 and MDA-MB-468 cell number compared to single of dual SHP2i/PI3Ki treatments (Fig. 3F, G, Fig S4E, F). We then assessed the effects of the triple combination on animal survival. Mice in the adjuvant setting were treated for 16 days by alternating 4 days of treatment with a 2-day break in order to limit drug toxicity. The Sunitinib single-agent and the CLR457/SHP099 dual-inhibition groups showed similar survival, with medians of 20 days and 23 days, respectively. The survival of the triple-combination group was significantly longer than any other group (29.5 days) (Fig. 3H). Altogether our results demonstrate the potential therapeutical benefits of co-targeting SHP2 and the PI3K pathways together with PDGFRβ in TNBC, as this increased overall survival in pre-clinical models.

## Discussion

Targeting signaling molecules in combination with chemo- or hormone therapy has improved the survival of patients at different stages of breast cancer. Approved mechanism-based therapies for breast cancer, such as Trastuzumab, Everolimus, Olaparib, Palbociclib, Ribociclib, Abemaciclib, and Alpelisib, target specific cancer dependencies with tolerable systemic effects [30]. Despite their initial success, these strategies are hindered by the development of resistance that leads to cancer cell insensitivity and restricts therapeutic solutions [31–34]. However, it is anticipated that combinations of therapies will potentiate the initial inhibition, thus overcoming resistance and improving patient outcome. Therefore, identifying optimal and tolerable combinations of therapies, particularly for metastatic disease, by assessing their effect on metastasis and overall survival in preclinical models will be paramount for the development of clinical trials. In the present study, we assessed the effects of SHP2 and/or PI3K inhibition in metastatic TNBC and found that dual inhibition decreases lung metastasis and enhances overall survival in this model.

The comparison of treatments with single agents and their effects on primary tumor growth, metastasis and overall survival was also informative, and showed differential sensitivity of cancer cells to targeted therapy at the primary site and in the lungs. Thus, primary tumor shrinkage alone as a measure of drug efficacy can be misleading. First, whereas PI3K inhibition had no effect on primary tumor growth, it increased the size of lung metastatic foci. We previously reported similar results using a dual PI3K/mTOR inhibitor in orthotopic TNBC models [24]. The discrepancy between effects on primary tumor growth and the consequences for metastasis has received experimental support in numerous publications [35–37], and our previous research also showed that CCL2, JAK2 or IL-8 inhibition has no effect on primary tumor growth but decreases lung metastases and increases overall survival [23, 24, 38]. Second, while SHP2 inhibition alone decreased primary tumor volume and lung metastases, it failed to improve animal survival. This observation may be explained either by an overshoot of metastases after cessation of treatment as we observed with anti-CCL2 treatment [23] or by metastases in other organs. Altogether, these findings stress the need for thorough preclinical evaluation of drug efficacy, not only in vitro and on primary tumor growth but also on metastasis and overall survival.

Protein and transcriptome analyses revealed activation of PI3K and ERK/MAPK pathways upon SHP2 blockade in a PDGFRβ-dependent manner and that this is blunted by combined inhibition of PI3K and SHP2. PDGFRs are RTKs that promote disease progression and therapy resistance in multiple cancer types, such as gliomas and colorectal cancer, and their exact activity in different breast cancer subtypes is currently being investigated. Indeed, PDGFRs can block tamoxifen action in ERα ( +) breast cancer by activating growth factor signaling and promoting ERα ligand-independent activation [39]. PDGFRβ blockade was also reported to dampen BRCA1-mediated tumorigenesis in a transgenic mouse model of breast cancer [40]. In our present study, we show that PDGFRβ acts as a rheostat for PI3K signaling upon SHP2i. Of note, SHP2 has been described previously to bind ligand-induced p-PDGFRβ and induce its dephosphorylation [41]. Our data corroborate this observation in TNBC models, where SHP2i led to accumulation of p-PDGFRβ, enabling p85 recruitment and subsequent activation of PI3K pathway signaling. Finally, we show that PDGFRβ inhibition significantly improved animal survival, either as single agent or in triple combination with SHP2i/PI3Ki, thus providing a rationale for combining these agents in clinical studies.

While multiple PI3K inhibitors are currently in clinical trials on solid tumors [42], an alpha-specific PI3K inhibitor has been approved for use, in combination with an endocrine therapy, to treat hormone receptor-positive (HER2)‑negative advanced breast cancer [43]. This pathway is also hyperactivated in the highly aggressive subtype TNBC, but the exact combination therapy that would enhance the efficacy of PI3K inhibition in the context of metastatic disease is not known. In addition, it has been reported that SHP2i can circumvent PI3K resistance in luminal and HER2-enriched breast cancer cells [44]. We show here that PI3K inhibition circumvents SHP2i resistance but, given the pleiotropic activity of SHP2, additional mechanisms of resistance may be at play. It has been reported for example that resistance to SHP099 therapeutic pressure in cancer cells can occur via activation of FGFR [45]. Indeed, co-targeting of SHP2 using SHP099 and FGFR using FIIN4 inhibitor was shown to decrease primary tumor and metastatic outgrowth in HER2-enriched and TNBC models. SHP2 was also identified as a key factor that promotes MEK/ERK pathway activation and therapy resistance in ALK and KRAS mutant non-small-cell lung cancers (NSCLC) [33, 34]. Our results provide a rationale for the use of dual SHP2 and PI3K inhibition in TNBC models, as this reduces primary tumor, metastases outgrowth and increases overall survival in preclinical models.

## Materials and methods

### Compounds

CLR457 and SHP099 were obtained from Novartis (Basel, Switzerland and Cambridge, USA), and Sunitinib from Pfizer Inc (Sutent). Compounds were prepared as 10 mM stock solutions in DMSO and stored protected from light at –20 °C. CLR457 (20 mg/kg), SHP099 (100 mg/kg) and Sunitinib (60 mg/kg) were freshly formulated in methylcellulose / Tween-80 (0.5% / 0.5%) and administered to mice by oral gavage at 5 ml/kg.

### Animal experiments

All in vivo experiments were performed in accordance with the Swiss animal welfare ordinance and approved by the cantonal veterinary office Basel Stadt. Female severe combined NOD-scid IL2rγnull (NSG) and Balb/c animals were maintained in the Friedrich Miescher Institute for Biomedical Research and the University Department of Biomedicine animal facilities in accordance with Swiss guidelines on animal experimentation. For orthotopic engraftment of cell lines, 0.3 × 10^6^ 4T1, 2 × 10^6^ MDA-MB-436, and 2 × 10^6^ MDA-MB-468 cells were suspended in 50 µL PBS and injected into mammary fat pad number 4 of 8-week-old mice. Tumor-bearing mice were randomized based on tumor volume prior to the initiation of treatment, which started when average tumor volume was at least 80 mm^3^. CLR457 was administered twice a day and SHP099 once daily. Tumors were measured every 3 – 4 days and tumor volumes calculated by the formula 0.5 x (larger diameter) x (smaller diameter)^2^. End-point tumor sizes were analyzed for synergism using the formula AB/C < A/C x B/C, where C is tumor volume Vehicle, A is tumor volume compound 1, B is tumor volume compound 2, and AB is tumor volume combination [46]. For survival studies, day 0 corresponds to tumor removal and animals were sacrificed as soon as they showed any signs of distress (e.g., breathing disorders, weight loss, or immobility).

### Cells, cell culture and reagents

SUM159 were propagated in Nutrient Mixture F-12 supplemented with 5% fetal calf serum, 0.5 μg/ml hydrocortisone, and 10 μg/ml insulin (all from Sigma), 100 IU/ml penicillin, 100 μg/ml streptomycin and 100 μg/ml Normocin (InvivoGen). Balb/c tumor-derived mammary cancer lines 4T1 were propagated in DMEM, with 10% fetal calf serum (all from Sigma), 100 IU/ml penicillin, 100 μg/ml streptomycin and 100 μg/ml Normocin (InvivoGen). MCF10A-HER2/HER3 [4] were propagated in DMEM/F12 medium (Invitrogen) supplemented with 5% horse serum (Hyclone), 20 ng/ml EGF (Peprotech), 0.5 μg/ml hydrocortisone, 100 ng/ml cholera toxin, and 10 μg/ml insulin (all from Sigma), 100 IU/ml penicillin, 100 μg/ml streptomycin and 100 μg/ml Normocin (InvivoGen). All other cell lines were obtained from and were cultured according to the protocols of the American Type Culture Collection. Profiling of human cell lines used highly-polymorphic short tandem repeat *loci* sequencing(STRs) (Microsynth). For treatment with inhibitor(s), cells were synchronized with 0.5% serum for 6 h to avoid masking effects of growth factors present under full-serum conditions.

### Cell number assay

Different cell lines were cultured overnight in 96-well plates at 1,000 to 5,000 cells/well before culture medium containing 0.5% FCS (or HS) and the inhibitor(s) described above were added. The culture medium with inhibitor(s) was renewed 48 h after initial treatment and cells were fixed 24 h later. Cell fixation, staining and quantification were performed using the Sulforhodamine B colorimetric assay. Values measured using the Sulforhodamine B colorimetric assay were analyzed for synergism using the formula AB/C < A/C x B/C, where C is value in DMSO condition, A is the value of inhibitor 1, B is the value of inhibitor 2, and AB is the inhibitors combination value.

### Immunoblotting and Phospho-RTK arrays

Cells were lysed with RIPA buffer (50 mM Tris–HCl pH 8, 150 mM NaCl, 1% NP-40, 0.5% sodium deoxycholate, 0.1% SDS) supplemented with 1 × protease inhibitor cocktail (Complete Mini, Roche), 0.2 mM sodium orthovanadate, 20 mM sodium fluoride and 1 mM phenylmethylsulfonyl fluoride. Lysates from xenografts were prepared by lysing kryo-homogenized tumor powder in RIPA buffer. Whole cell lysates (30–80 μg) were subjected to SDS-PAGE, transferred to PVDF membranes (Immobilon-P, Millipore) and blocked for 1 h at room temperature with 5% milk in PBS-0.1% Tween-20. Membranes were then incubated overnight with primary antibodies as indicated and exposed to secondary HRP-coupled anti-mouse or anti-rabbit antibodies at 1:7,500 for 2 h at room temperature. The following antibodies were used: anti-pAKT (Ser473, Cell Signaling, #4060), anti-pERK1/2 (Thr202/Tyr204, Cell Signaling, #4377), anti-pPDGFRβ (Tyr751, Thermo Scientific, MA-14823), anti-ERK2 (Santa Cruz, sc-1647), anti-pS6 (Ser235/236, Cell Signaling, #2211), anti-PDGFRβ (Cell Signaling, #3169), anti-AKT (Santa Cruz, sc-5298) and anti-Vinculin (Thermo Scientific, #14–9777-80). Phospho-RTK arrays on tumor lysates were performed using the Proteome Profiler Mouse Phospho-RTK Array Kit (R&D systems) according to the manufacturer’s protocol.

### Co-immunoprecipitation

4T1 cells were plated in 10 cm dishes, starved for 6 h in 1% Serum medium and treated for 24 h with DMSO or SHP099 at 5 μM. Cells were washed in ice-cold PBS and lysed in RIPA Buffer (50 mM Tris–HCl pH 8, 150 mM NaCl, 1% NP-40, 0.5% sodium deoxycholate, 0.1% SDS) supplemented with 1 × protease inhibitor cocktail (Complete Mini, Roche), 0.2 mM sodium orthovanadate, 20 mM sodium fluoride and 1 mM phenylmethylsulfonyl fluoride, for 30 min at 4 °C. Cell lysates were sonicated and protein abundance was quantified. Input fraction was recovered and 500 μg of proteins were subjected to immuno-precipitation with 2 μg of anti-p85 antibodies (Cell Signaling, #4257) or 2 μg of control IgG (Cell Signaling, #3900), and 100 μl of binding buffer (Thermo Scientific, #10007D). After overnight incubation at 4 °C on a turning wheel, 50 ul of protein A magnetic beads (Dynabeads, Thermo Scientific, #10002D) was added to the mixture for 4 h more. Immune-complexes were washed 3 times using washing buffer (Thermo Scientific, #10007D) on a magnetic rack, and extracted using 20 μl of RIPA buffer supplemented with 2 × Laemmli buffer. Input and immune-complexes fractions were subjected to proteins analysis by western-blot using anti-p85 and anti-PDGFRβ antibodies.

### Proximity ligation assay

The DUOLink in situ Detection Reagent Red kit (Sigma – DUO92008) was used to detect and quantify PDGFRβ and p85 interactions. DMSO- or SHP099-treated 4T1 and MDA-MB-468 cells seeded on glass coverslips (24 well-plate) were fixed in 4% PFA for 15 min at RT, permeabilized with PBS 0.1% Triton for 30 min at RT and blocked with Duolink blocking buffer for 1 h at 37 °C. Cells were then incubated overnight at 4 °C with primary antibodies directly coupled to the DUOLink DNA probes using the in situ Probemaker PLUS kit (Sigma – DUO92009) diluted in DUOLink Probemaker PLA probe diluent at 1/100. The PLA reaction was then performed according to the manufacturer’s instructions. Briefly, cells were washed in buffer A (provided) and the two steps of ligation of the DNA probe and rolling-circle amplification (RCA) were carried out for 30 min and 100 min respectively in a humid dark chamber at 37 °C. Cells were then washed in buffer B (provided) and the coverslips mounted on slides using the Duolink Mounting Medium with DAPI. Z-stacked images were acquired with a Nikon Ti2 microscope at 40 × magnification using Dapi and 555 channels. Images were analyzed using the FIJI software and represented as a number of dots (interactions) per cell, with hundreds of cells analyzed per experiment.

### Immunohistochemistry

Tumors were fixed in 10% neutral buffered formalin (NBF) for 24 h at 4 °C, washed with 70% EtOH, and embedded in paraffin. Sections of 2.5 µm were cut and processed for hematoxylin and eosin (H&E) staining and immunohistochemistry. Prior to fixation, dissected lungs were inflated by injecting 5 mL of PBS through the trachea, then inflated with 5 mL of 10% NBF and gently released into a tube filled with 10% NBF. Immunohistochemical staining was performed on formalin-fixed, paraffin-embedded tissue sections using a Discovery XT (Ventana) fully automated system for anti-pAKT (Ser473), anti-pERK1/2 (Thr202/Tyr204) and anti-cleaved Caspase-3 (Asp175). Algorithms for quantitative analysis of immunostained-positive areas and the areas of lung metastases were designed in Halo software that allowed assessment of the relative fractions of positive areas.

### Apoptosis assay

Cells were synchronized with DMEM 0.5% serum overnight and then supplemented with medium containing inhibitor(s). Fresh inhibitors were added after 48 h and cells (floating and adherent) were collected 24 h later using trypsin–EDTA, resuspended in growth medium and counted. For Annexin V/propidium iodide staining, cells were washed twice with cold Cell Staining Buffer (BioLegend, #420,201) and resuspended in Annexin V Binding Buffer (BioLegend, #422,201) at a concentration of 1 × 10^6^ cells/mL. Aliquots of Alexa Fluor 647 Annexin V (5 µL) (BioLegend, #640,911) and of propidium iodide (10 µL) (BioLegend, #421,301) were added to 100 µL of this suspension, which was then incubated for 15 min at room temperature in the dark. After addition of 400 µl of Annexin V Binding Buffer to each tube, samples were analyzed by flow cytometry.

### Transcriptomic analysis

Total RNA was extracted from frozen tumors using the RNeasy Plus Mini Kit (Qiagen, #74,136) and sample quality was controlled on an Agilent 2100 Bioanalyzer system with the RNA6000 Nano kit (Agilent, #5067–1511). mRNA isolation was performed with the NEBNext Poly(A) mRNA magnetic isolation module (NEB, #E7490) and libraries were prepared with the NEBNext Ultra II Directional RNA Library Prep kit (NEB, #E7765) according to the manufacturer’s recommendations. Samples were individually barcoded during library preparation using NEBNext Multiplex Oligos for Illumina Index Primers Sets 1 and 2 (NEB, #E7335 and #E7500). Library quality control was performed with the DNA1000 kit (Agilent, #5067–1504) on the Agilent 2100 Bioanalyzer system. Finally, libraries were sequenced on an Illumina NextSeq 500 that generated paired-end 75-bp reads. Adaptor trimming was performed using cutadapt. Trimmed reads were aligned to the GRCm38 genome using the two-pass approach of STAR. A median of 53 million reads (range 44–60) were aligned per sample. qCount from QuasR was then used to obtain counts at the gene level. Differential gene expression analysis was performed using edgeR [47]. A cutoff of log2 fold change > 1 and adjusted to *P* < 0.05 (corrected by the Benjamini–Hochberg algorithm method) was applied to selected genes. Network analysis was performed on the cBioportal website using the breast cancer dataset METABRIC [48].

### 3D 4T1 cell culture

For in vitro drug treatments, 4T1 cells were seeded in DMEM containing 10% FCS and 30% Matrigel Growth Factor Reduced (Corning, 356,231) at 300 cells per well in 384-well plates in quadruplicates. After three days, 3D colonies were treated with DMEM containing 1% FCS together with CLR457, SHP009, Sunitinib or combinations thereof at the indicated concentrations. Two days later, 50% of the culture medium was exchanged with medium containing drugs at 200% higher concentrations. Cells were kept under treatment for a further two days. At treatment day 5, the viability of cells was assessed by the CellTiter-Glo 3D Cell Viability Assay (Promega, G9618) according to the manufacturer’s instructions. In brief, after removing the culture medium, cells were lysed in 25 µl CellTiter-Glo 3D Reagent. After a 30-min incubation at room temperature on a horizontal shaker, luminescence was recorded for 0.5 s with an ELISA-reader.

### Statistical analysis

Unless stated differently in the figure legends, all results shown represent at least three independent experiments and are reported as means ± STDEV. Data were tested for normal distribution and ANOVAs tests were applied. GraphPad Prism 7.04 was used for Kaplan–Meier survival analysis and log rank Mantel-Cox tests were applied to test statistical significance (SAS), as well as for all other statistical tests (SAS). The *P* values < 0.05 were considered statistically significant.

### Data deposition

Transcriptomic data are available on the GEO database, reference GSE128051 (https://www.ncbi.nlm.nih.gov/geo/query/acc.cgi?acc=GSE128051).

## Supplementary Information

**Additional file 1: Figure S1.** Mutation profiles and baseline phosphorylation of S6, AKT and ERK1/2 kinases in a panel of breast cancer cell lines. **A** Table depicting the mutation status of a panel of breast cancer cell lines for the indicated oncogenes and tumor suppressors, inferred from the DepMap (https://depmap.org/portal/) and COSMIC (https://cancer.sanger.ac.uk/cosmic) databases. Blank spaces indicate no referenced mutations or genetic alterations. **B** Western blots showing p-S6 (S235/236), p-AKT (S473) and pERK1/2 (T202/Y204) across the panel of indicated breast cancer cell lines. 40 µg of proteins were loaded and total ERK2 and Vinculin were used as loading control. **C** Quantification (using FIJI) of p-AKT (S473) and pERK1/2 (T202/Y204). Cell lines were ranked in a descending order, based on their abundance for the indicated phospho-site.

**Additional file 2: Figure S2.** SHP2 single inhibition enhances apoptosis and PI3K/SHP2 dual inhibition prolongs overall survival of TNBC-tumor-bearing mice. **A** Cell numbers of 4T1 cells treated with CLR457 (PI3Ki) or SHP099 (SHP2i) at the indicated concentrations for 72 h. *n* = 3 biological replicates. ** *P* ≤ 0.01, *** *P *≤ 0.001, **** *P* ≤ 0.0001; One-way ANOVA test. Data shown are means of cell numbers ± STDEV. **B** SRB assays in the MDA-MB-468 model establishing IC50 values for SHP099 and CLR457 treatment. n = 2 biological replicates with 4 technical replicates. Data shown are means ± STDEV. **C** Cell numbers of 4T1 cells treated with CLR457 (PI3Ki) and SHP099 (SHP2i) at the indicated concentrations for 72 h. *n* = 3 biological replicates. *** *P* ≤ 0.001; One-way ANOVA test. Data shown are means of cell numbers ± STDEV. **D** Representative FACS plots of annexin V (AV) / propidium iodide (PI) apoptosis analysis of 4T1 cells treated for 3 days with CLR457 (top panel) or SHP099 (bottom panel) at the indicated concentrations. Fresh inhibitors were added after 48 h. Quantification is shown as bar graph. **E** Representative FACS plots of annexin V (AV) / propidium iodide(PI) apoptosis analysis of 4T1 cells treated with CLR457 and SHP099 for 3 days (top panel). Fresh inhibitors were added after 48 h. Quantification is shown as a bar graph (bottom panel). *n* = 3 biological replicates. * *P* ≤ 0.05, *** *P *≤ 0.001; Two-way ANOVA test. Data shown are means ± STDEV. **F** Tumor volume and overall survival of MDA-MB-468 tumor-bearing mice treated as described (adjuvant settings). *n* = 4 – 6 animals. * *P* ≤ 0.05, ** *P* ≤ 0.01; One-way ANOVA test. Data shown are mean tumor volumes ± SEM. **G** Left panel: Representative images of H&E-stained lungs from 4T1 tumor-bearing mice treated as described in Fig. 1G. Black lines delineate metastases. Scale bar 100 µm. Right panel: Bar graph of the percentages of lungs with metastases from Vehicle-, CLR457-, SHP099-, and CLR457 + SHP099-treated groups. *n* = 7 – 8 animals.*** *P* ≤ 0.001, **** *P* ≤ 0.0001; One-way ANOVA test. Data shown are means ± STDEV. **H** Bar graph of the quantification of metastatic areas in Vehicle- and CLR457-treated groups (the data for SHP099 and CLR457 + SHP099 groups are not shown because no metastatic foci were detected). Small 0-0.1; Medium 0.1-0.5; Large > 0.5 mm^2^. *n* = 7 – 8 animals. **** *P* ≤ 0.0001; Student t-test. Data shown are means ± STDEV.

**Additional file 3: Figure S3.** Dual and single inhibitions of PI3K and SHP2 block the activation of the PI3K and MAPK pathways *in vivo. ***A** Representative images of p-AKT(Ser473) (left panel) and p-ERK (Thr202/Tyr204) (middle panel) IHC-stained 4T1 tumors from mice treated for 4 days as indicated. Bar graphs show quantification using the pixel count algorithm performed with Halo software (right panels). Tumors were collected as described in Fig. 1D. Scale bar 100 µm. Data shown are means ± STDEV. *n* = 4 - 5, *P ≤ 0.05; One-way ANOVA test. **B** Principal Component Analysis (PCA) plot of RNA-seq data from 4T1 tumors of mice treated with Vehicle (Vhc), CLR457 and/or SHP099. **C** Venn diagram of differentially expressed genes (up and down regulated) in 4T1 tumors from mice treated with CLR457 (PI3Ki) and/or SHP099 (SHP2i) (3 h after the last treatment), as compared to the Vehicle group. *n* = 4 – 5 animals per group. LogFC > 1 or < -1, FDR < 0.05. **D** Volcano plots showing differentially expressed genes in 4T1tumors from mice treated as indicated. *n* = 4 – 5 animals, LogFC > 1, FDR < 0.05. **E** C2 curated genesets from GSEA functional annotation of genes down-regulated upon SHP2i as compared to Vehicle. LogFC < -1, FDR < 0.05. **F** WikiPathways 2021 functional annotation of genes down-regulated upon PI3Ki as compared to Vehicle. LogFC < -1, FDR < 0.05. **G** Heatmap of the top 35 upregulated genes in 4T1 tumors of mice treated with Vehicle or SHP099. *n* = 4 – 5 animals. LogFC > 1.5, FDR < 0.01.

**Additional file 4: Figure S4.** SHP2i increases PDGFRβ phosphorylation at Y751 and sensitizes cells to sunitinib treatment. **A** Volcano plots of transcriptomic variation of the “PDGF-related gene family” and the “top 35 upregulated genes” in 4T1 tumors from mice treated with SHP099. Data shown are individual values. *n *= 4 – 5 animals per group. LogFC > 1.5, FDR < 0.01. **B** Heatmap of PDGF-family-related genes in 4T1 tumors of mice treated with Vehicle, CLR457 and/or SHP099. Data shown are individual values. *n *= 4 – 5 animals per group. LogFC >1.5, FDR < 0.01. **C** Western blots showing p-PDGFRβ (Y751, p85 docking site) and p-ERK2 (T202/Y204) upon short term (30 min) and long term (24 h) SHP099 treatment (10 µM) in the MDA-MB-468 cell line. Total ERK2 and PDGFRβ were used as loading control. Quantification normalized to DMSO is shown. **D** Proximity Ligation Assay of PDGFRβ/p85 in MDA-MB-468 cells starved in DMEM 1 % FCS treated with DMSO or SHP2i (SHP099, 10 µM). Quantification of dots (interactions) per cell and representative images are shown. Brightness and contrast for the red and blue channels have been adjusted identically for all images. *n* = 3 biological replicates. *** *P* < 0.001; Student’s t-test. Data shown are mean ± STDEV. Scale bar 20 µM. **E** SRB assays in the MDA-MB-468 model establishing IC50 value for SHP099 and CLR457 treatment. n = 2 biological replicate with 4 technical replicates. Data shown are mean ± STDEV. **F** SRB assay using the MDA-MB-468 cells treated with DMSO, SHP099 (5 µM), CLR457 (2 µM), Sunitinib (3 µM) and the indicated combinations, for 3 consecutive days. n = 2 biological replicates with 4 technical replicates. * *P* ≤ 0.05, *** *P* ≤ 0.001; One-way ANOVA test. Data shown are mean ± STDEV.
