# Supplementary material for: PI3K inhibition circumvents resistance to SHP2 blockade in metastatic triple-negative breast cancer
Source: J Mammary Gland Biol Neoplasia. 2023 Jun 9;28(1):13. doi: 10.1007/s10911-023-09539-9 (PMC10256672; doi:10.1007/s10911-023-09539-9)

A

	ZR-75-1	MDA-MB-231	T47D	SUM159PT	MCF7	BT474	HCC1954	4T1	MDA-MB-468	BT549	MDA-MB-436	MCF10A HER2/3
AKT1												
PIK3CA			H1047R	H1047L	E545K	K111N	H1047R					
PTEN	L108R								Frameshift	Frameshift		
KRAS		G13D										
BRAF		G464V										
HRAS	E162K			G12D								
MAPK1						H61Q						
ERBB2						Amplification	Amplification		Frameshift			Over-expression
EGFR												
TP53		R280K	L194F	Frameshift		E285K	Y163C	Frameshift	R273H	R249S	Frameshift	
BRCA1									M965I		Frameshift	
CDKN2A		Del										
RB1				K530N						Frameshift		
STAT3						F174L						
NFKB1						E195Q						
STAT4											Q193K	

B

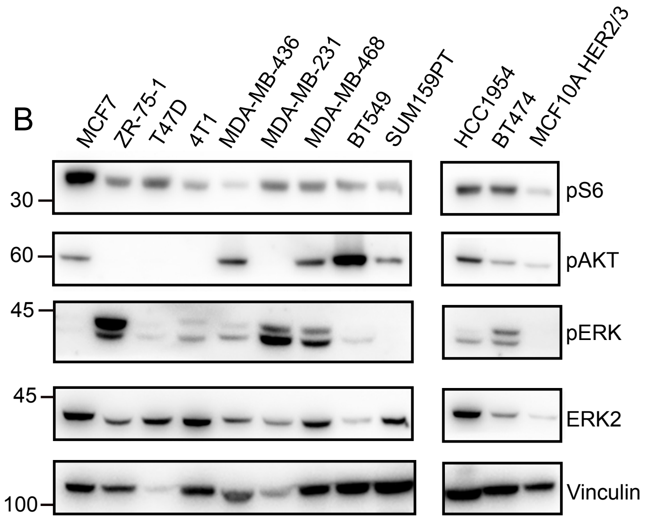

C

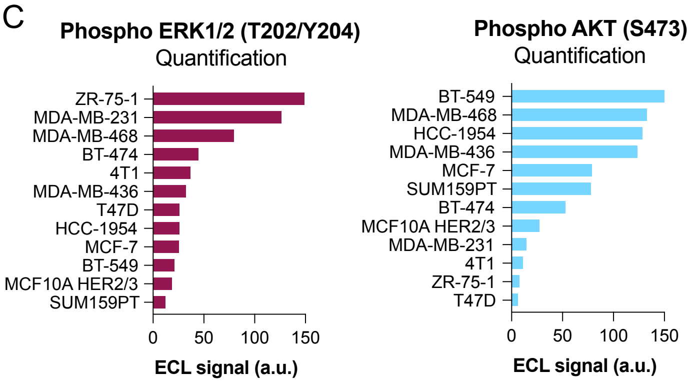

Supplement: Supplementary file 1 — Additional file 1: Figure S1. Mutation profiles and baseline phosphorylation of S6, AKT and ERK1/2 kinases in a panel of breast cancer cell lines. A Table depicting the mutation status of a panel of breast cancer cell lines for the indicated oncogenes and tumor suppressors, inferred from the DepMap (https://depmap.org/portal/) and COSMIC (https://cancer.sanger.ac.uk/cosmic) databases. Blank spaces indicate no referenced mutations or genetic alterations. B Western blots showing p-S6 (S235/236), p-AKT (S473) and pERK1/2 (T202/Y204) across the panel of indicated breast cancer cell lines. 40 µg of proteins were loaded and total ERK2 and Vinculin were used as loading control. C Quantification (using FIJI) of p-AKT (S473) and pERK1/2 (T202/Y204). Cell lines were ranked in a descending order, based on their abundance for the indicated phospho-site. [file 10911_2023_9539_MOESM1_ESM.pdf]
