# Supplementary figures and images for: PI3K inhibition circumvents resistance to SHP2 blockade in metastatic triple-negative breast cancer
Source: J Mammary Gland Biol Neoplasia. 2023 Jun 9;28(1):13. doi: 10.1007/s10911-023-09539-9 (PMC10256672; doi:10.1007/s10911-023-09539-9)

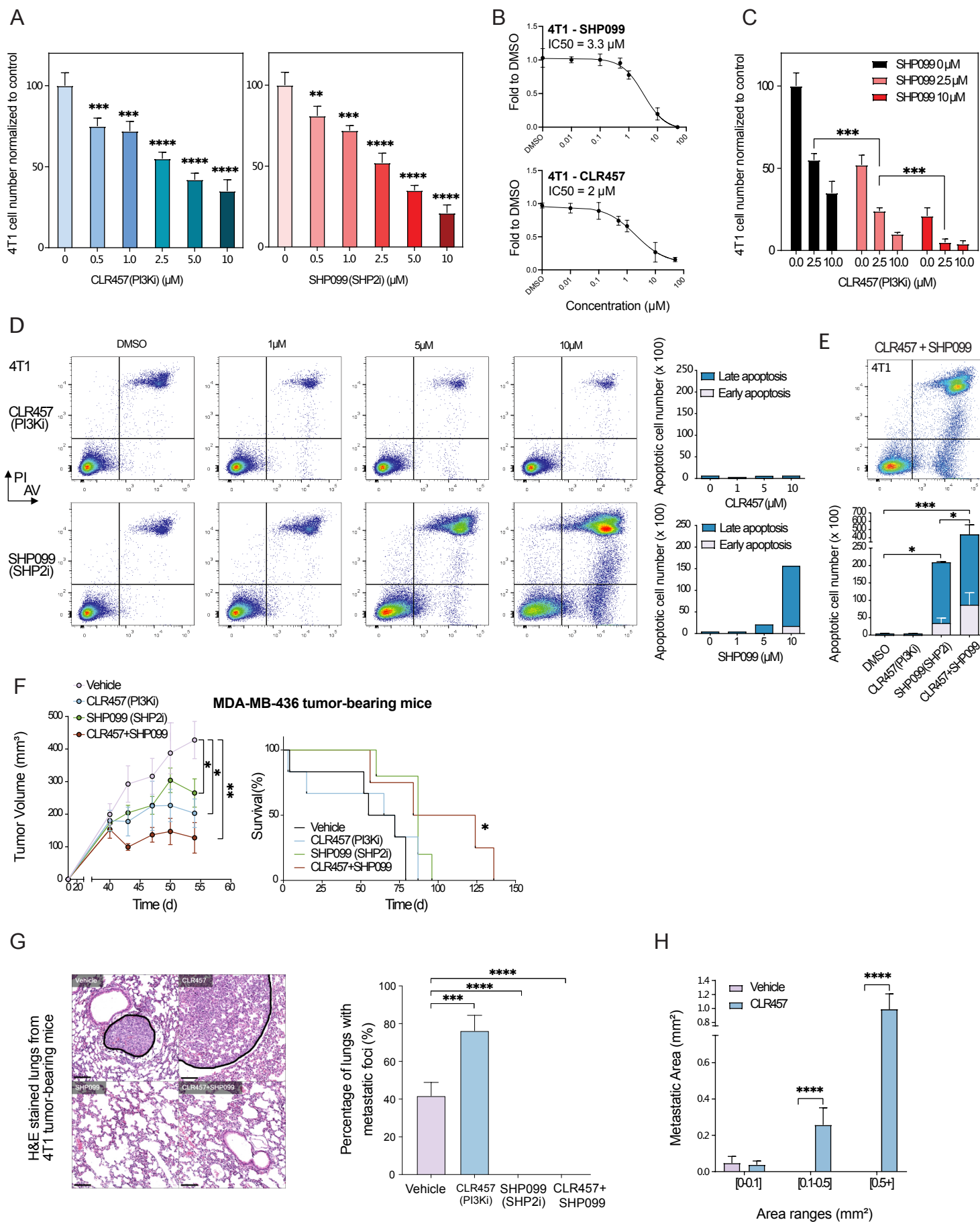

Supplement: Supplementary file 2 — Additional file 2: Figure S2. SHP2 single inhibition enhances apoptosis and PI3K/SHP2 dual inhibition prolongs overall survival of TNBC-tumor-bearing mice. A Cell numbers of 4T1 cells treated with CLR457 (PI3Ki) or SHP099 (SHP2i) at the indicated concentrations for 72 h. n = 3 biological replicates. ** P ≤ 0.01, *** P ≤ 0.001, **** P ≤ 0.0001; One-way ANOVA test. Data shown are means of cell numbers ± STDEV. B SRB assays in the MDA-MB-468 model establishing IC50 values for SHP099 and CLR457 treatment. n = 2 biological replicates with 4 technical replicates. Data shown are means ± STDEV. C Cell numbers of 4T1 cells treated with CLR457 (PI3Ki) and SHP099 (SHP2i) at the indicated concentrations for 72 h. n = 3 biological replicates. *** P ≤ 0.001; One-way ANOVA test. Data shown are means of cell numbers ± STDEV. D Representative FACS plots of annexin V (AV) / propidium iodide (PI) apoptosis analysis of 4T1 cells treated for 3 days with CLR457 (top panel) or SHP099 (bottom panel) at the indicated concentrations. Fresh inhibitors were added after 48 h. Quantification is shown as bar graph. E Representative FACS plots of annexin V (AV) / propidium iodide(PI) apoptosis analysis of 4T1 cells treated with CLR457 and SHP099 for 3 days (top panel). Fresh inhibitors were added after 48 h. Quantification is shown as a bar graph (bottom panel). n = 3 biological replicates. * P ≤ 0.05, *** P ≤ 0.001; Two-way ANOVA test. Data shown are means ± STDEV. F Tumor volume and overall survival of MDA-MB-468 tumor-bearing mice treated as described (adjuvant settings). n = 4 – 6 animals. * P ≤ 0.05, ** P ≤ 0.01; One-way ANOVA test. Data shown are mean tumor volumes ± SEM. G Left panel: Representative images of H&E-stained lungs from 4T1 tumor-bearing mice treated as described in Fig. 1G. Black lines delineate metastases. Scale bar 100 µm. Right panel: Bar graph of the percentages of lungs with metastases from Vehicle-, CLR457-, SHP099-, and CLR457 + SHP099-treated groups. n = 7 – 8 animals.* [file 10911_2023_9539_MOESM2_ESM.pdf]

A

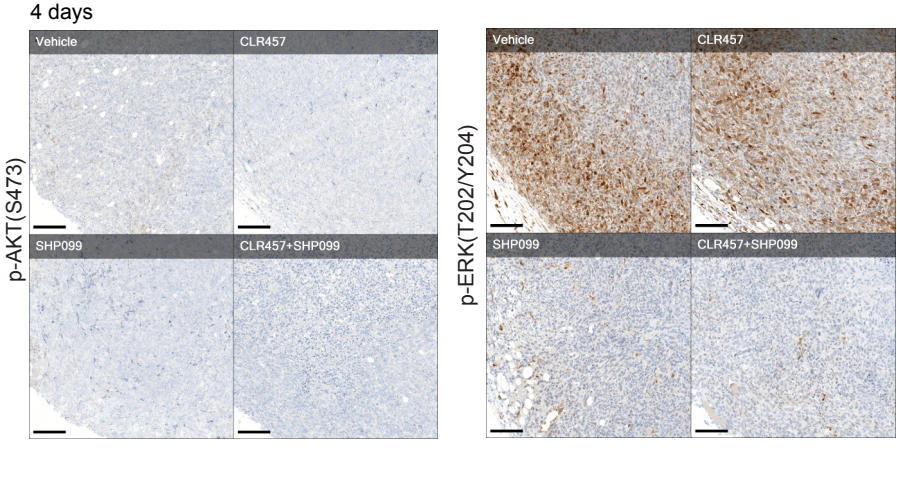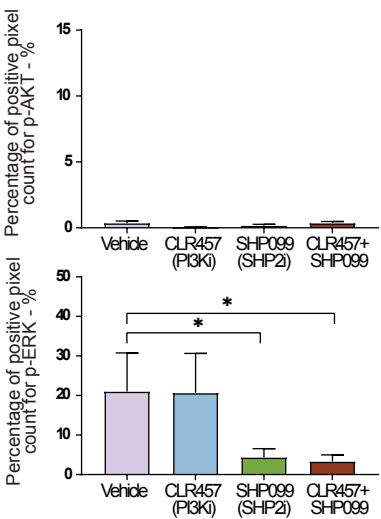

B

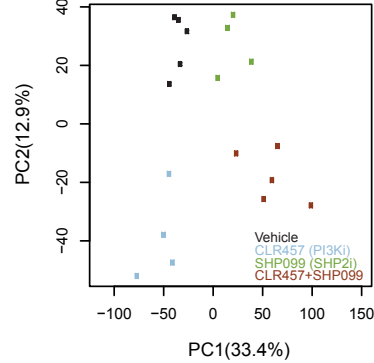

C

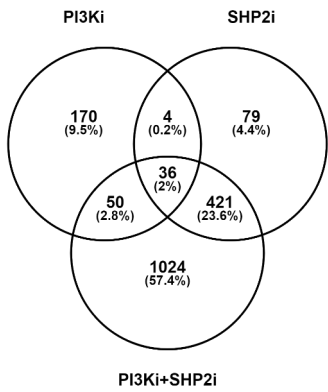

E

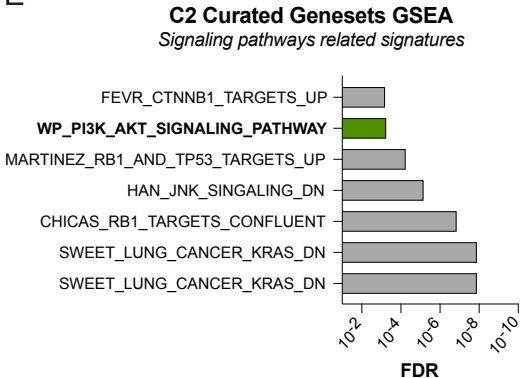

D

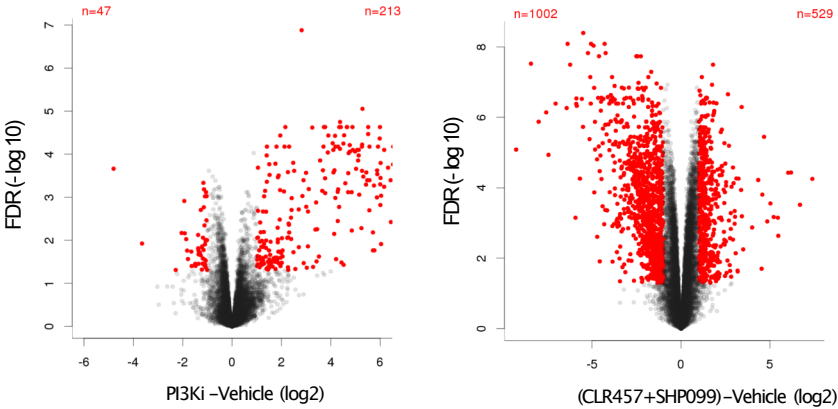

F

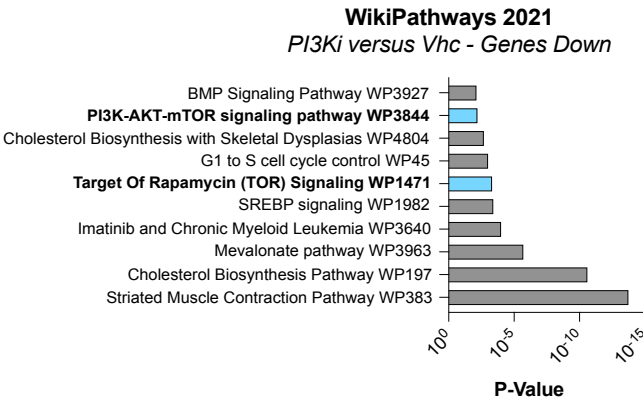

G

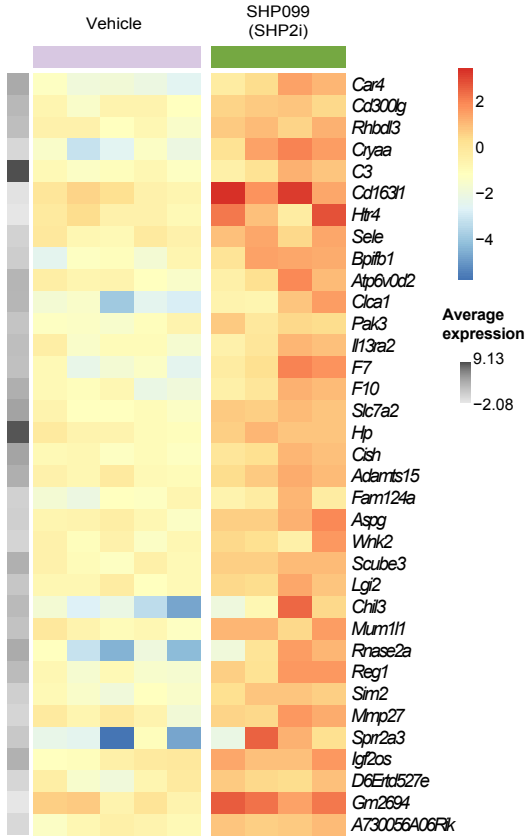

Supplement: Supplementary file 3 — Additional file 3: Figure S3. Dual and single inhibitions of PI3K and SHP2 block the activation of the PI3K and MAPK pathways in vivo. A Representative images of p-AKT(Ser473) (left panel) and p-ERK (Thr202/Tyr204) (middle panel) IHC-stained 4T1 tumors from mice treated for 4 days as indicated. Bar graphs show quantification using the pixel count algorithm performed with Halo software (right panels). Tumors were collected as described in Fig. 1D. Scale bar 100 µm. Data shown are means ± STDEV. n = 4 - 5, *P ≤ 0.05; One-way ANOVA test. B Principal Component Analysis (PCA) plot of RNA-seq data from 4T1 tumors of mice treated with Vehicle (Vhc), CLR457 and/or SHP099. C Venn diagram of differentially expressed genes (up and down regulated) in 4T1 tumors from mice treated with CLR457 (PI3Ki) and/or SHP099 (SHP2i) (3 h after the last treatment), as compared to the Vehicle group. n = 4 – 5 animals per group. LogFC > 1 or < -1, FDR < 0.05. D Volcano plots showing differentially expressed genes in 4T1tumors from mice treated as indicated. n = 4 – 5 animals, LogFC > 1, FDR < 0.05. E C2 curated genesets from GSEA functional annotation of genes down-regulated upon SHP2i as compared to Vehicle. LogFC < -1, FDR < 0.05. F WikiPathways 2021 functional annotation of genes down-regulated upon PI3Ki as compared to Vehicle. LogFC < -1, FDR < 0.05. G Heatmap of the top 35 upregulated genes in 4T1 tumors of mice treated with Vehicle or SHP099. n = 4 – 5 animals. LogFC > 1.5, FDR < 0.01. [file 10911_2023_9539_MOESM3_ESM.pdf]

A

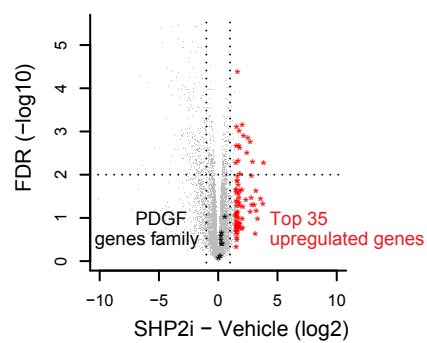

B

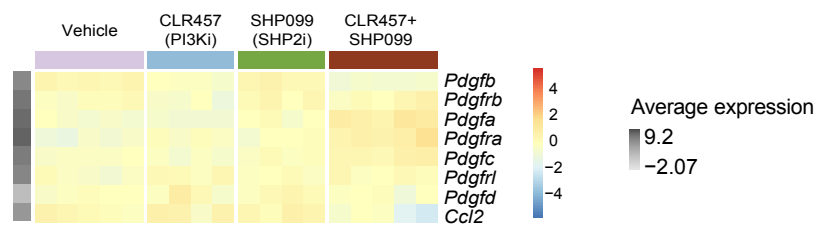

C

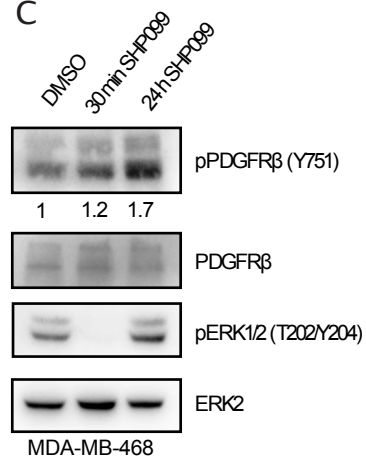

D

PLA - PDGFRβ/p85

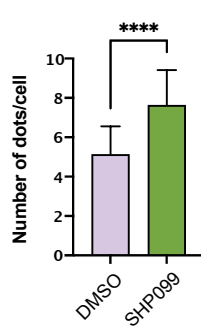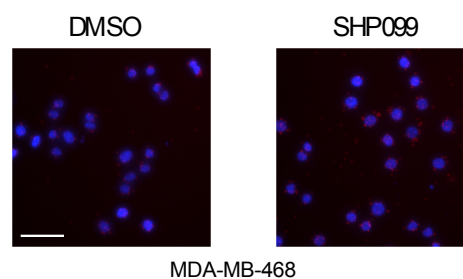

MDA-MB-468

E

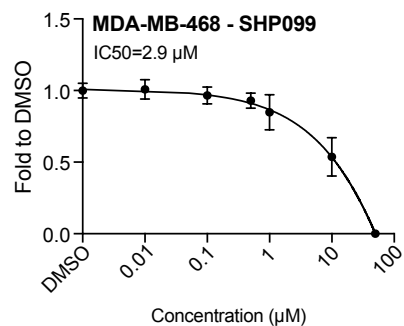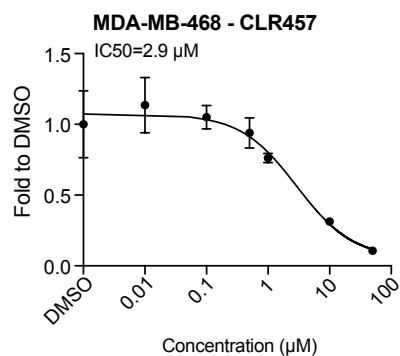

F

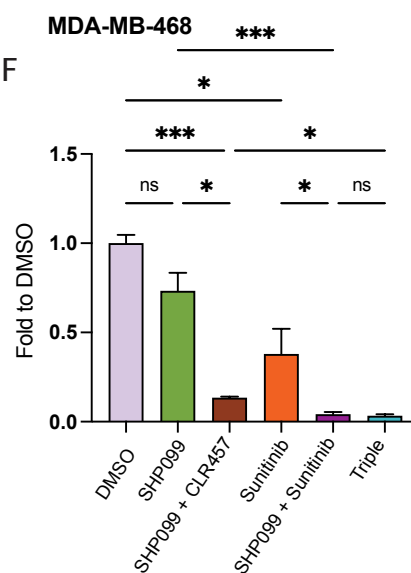

Supplement: Supplementary file 4 — Additional file 4: Figure S4. SHP2i increases PDGFRβ phosphorylation at Y751 and sensitizes cells to sunitinib treatment. A Volcano plots of transcriptomic variation of the “PDGF-related gene family” and the “top 35 upregulated genes” in 4T1 tumors from mice treated with SHP099. Data shown are individual values. n = 4 – 5 animals per group. LogFC > 1.5, FDR < 0.01. B Heatmap of PDGF-family-related genes in 4T1 tumors of mice treated with Vehicle, CLR457 and/or SHP099. Data shown are individual values. n = 4 – 5 animals per group. LogFC >1.5, FDR < 0.01. C Western blots showing p-PDGFRβ (Y751, p85 docking site) and p-ERK2 (T202/Y204) upon short term (30 min) and long term (24 h) SHP099 treatment (10 µM) in the MDA-MB-468 cell line. Total ERK2 and PDGFRβ were used as loading control. Quantification normalized to DMSO is shown. D Proximity Ligation Assay of PDGFRβ/p85 in MDA-MB-468 cells starved in DMEM 1 % FCS treated with DMSO or SHP2i (SHP099, 10 µM). Quantification of dots (interactions) per cell and representative images are shown. Brightness and contrast for the red and blue channels have been adjusted identically for all images. n = 3 biological replicates. *** P < 0.001; Student’s t-test. Data shown are mean ± STDEV. Scale bar 20 µM. E SRB assays in the MDA-MB-468 model establishing IC50 value for SHP099 and CLR457 treatment. n = 2 biological replicate with 4 technical replicates. Data shown are mean ± STDEV. F SRB assay using the MDA-MB-468 cells treated with DMSO, SHP099 (5 µM), CLR457 (2 µM), Sunitinib (3 µM) and the indicated combinations, for 3 consecutive days. n = 2 biological replicates with 4 technical replicates. * P ≤ 0.05, *** P ≤ 0.001; One-way ANOVA test. Data shown are mean ± STDEV. [file 10911_2023_9539_MOESM4_ESM.pdf]
